# Relationship between Animal Sourced Food Consumption and Early Childhood Development Outcomes

**DOI:** 10.3390/nu15020315

**Published:** 2023-01-09

**Authors:** Ashish Pokharel, Patrick Webb, Laurie C. Miller, Sonia Zaharia, Robin Shrestha, Dale Davis, Johanna Andrews Trevino, Kedar Prasad Baral, Krishna Paudel, Shibani Ghosh

**Affiliations:** 1 Helen Keller International, Lalitpur 44600, Nepal; 2 Feed the Future Innovation Lab for Nutrition, Lalitpur 44600, Nepal; 3 Friedman School of Nutrition Science and Policy, Tufts University, Boston, MA 02111, USA; 4 School of Public Health, Patan Academy of Health Sciences, Lalitpur 44600, Nepal; 5 Kanti Children’s Hospital, Kathmandu 44600, Nepal

**Keywords:** early childhood development, physical growth, animal sourced food, diet, Nepal

## Abstract

Early-childhood development (ECD) is an important determinant of a child’s cognitive ability, learning, productivity, and lifetime earnings. Animal-sourced food (ASF), which is a rich source of high-quality protein and micronutrients, has been linked with ECD outcomes. This study investigates the relationship between the number, frequency, and cumulative consumption of ASF at 6, 9, 12, and 18 months of age and ECD outcomes at 24 months of age, controlling for physical growth. The study uses data collected from 701 mother–child pairs from an observational birth cohort study carried out in Banke, Nepal. ECD outcomes were assessed through a standardized ages and stages questionnaire (ASQ-3) tool. Separate multivariable ordinary least squares regression models were used to test for associations. Significant positive association was seen between total ASQ-3 score at 24 months of age and any ASF consumption at 18 months (β = 8.98, *p*-value < 0.01), controlling for growth outcomes. The study findings highlight the positive contribution and the accumulating benefit of consistent ASF consumption on ECD outcomes. This study recommends support and promotion of ASF intake among young children in Nepal through policy and programming actions relevant to female education; nutrition knowledge; quality ASF production; improved market access; cold storage; and poverty reduction.

## 1. Introduction

The importance of early-childhood development (ECD) as a determinant of children’s educational attainment and lifetime earnings has long been emphasized in the literature [1,2]. The first few years of life are critical periods of brain development, having long-term effect on the brain’s plasticity for learning and cognitive functioning [3,4]. It is estimated that in low-and-middle income countries, about 219 million (39%) children under the age of 5 years fail to reach their cognitive development potential, leading to an average deficit of almost 20% in adult annual income [5].

The exact relationship between growth and ECD has not been well established as both are affected by common factors such as diet, childcare, community, and infectious diseases, indicating that their association may be a co-occurrence, causal, or a combination, i.e., both causal and co-occurrence [6,7]. Despite this ambiguity, several studies have shown that ECD and child’s physical growth are interconnected [8,9,10,11,12]. Gains in height/length were found to be positively associated with cognitive development [13,14]. Head circumference, an indicator of brain volume, has been indirectly associated with ECD outcomes [15,16,17]. Similarly, studies have shown significant associations between stunting and later life cognitive deficits [13,18,19]. As physical growth is interconnected with ECD, it is often used as a proxy for measuring ECD, despite growth not capturing specific cognitive, motor, language, and social domains of ECD. Thus, assessing ECD along with physical growth is necessary to obtain a better picture of a child’s overall development.

Several studies have investigated the role of animal-sourced foods (ASF) consumption on ECD and physical growth. ASF, mainly, milk, meat, eggs, and fish, are of particular interest because they are rich sources of high-quality protein, essential amino acids, and micronutrients and have been linked with ECD and physical growth outcomes [20,21]. Several studies have observed positive associations between ASF consumption and ECD indicators such as test scores and standardized developmental testing scores [22,23,24,25]. Studies have shown a positive relationship between ASF consumption and head circumference for age z-scores (HCZ) [26,27,28]. Likewise, studies have shown a positive association between the number of ASF consumed and a lowered risk of stunting [21,29,30,31] and length-for-age Z-scores (LAZ) [30,32,33].

Our main objective in this study is to understand the relationship between ASF consumption and ECD outcomes while controlling for physical growth. Controlling for physical growth will help us better understand the relationship of ASF consumption with ECD outcomes, regardless of the nature of relationship between ECD outcomes and physical growth, which is still not fully understood. As a secondary objective, we also investigate the relationship between ASF consumption and physical growth outcomes. The novelty in this study is that we examine different dimensions of ASF consumption and its relationship with ECD outcomes, while controlling for physical growth outcomes. This is important as little is known on the relationship between ASF consumption in early life (from 6 months onwards and cumulative effect of ASF consumption) and ECD outcomes at 24 months. This paper tries to address these knowledge gaps. We hypothesize that the total number of ASFs consumed and ASF consumption frequency measured at 6, 9, 12, and 18 months of age as well as cumulative ASF consumption in this period are positively associated with ECD outcomes at 24 months of age.

## 2. Materials and Methods

**Figure A1:**
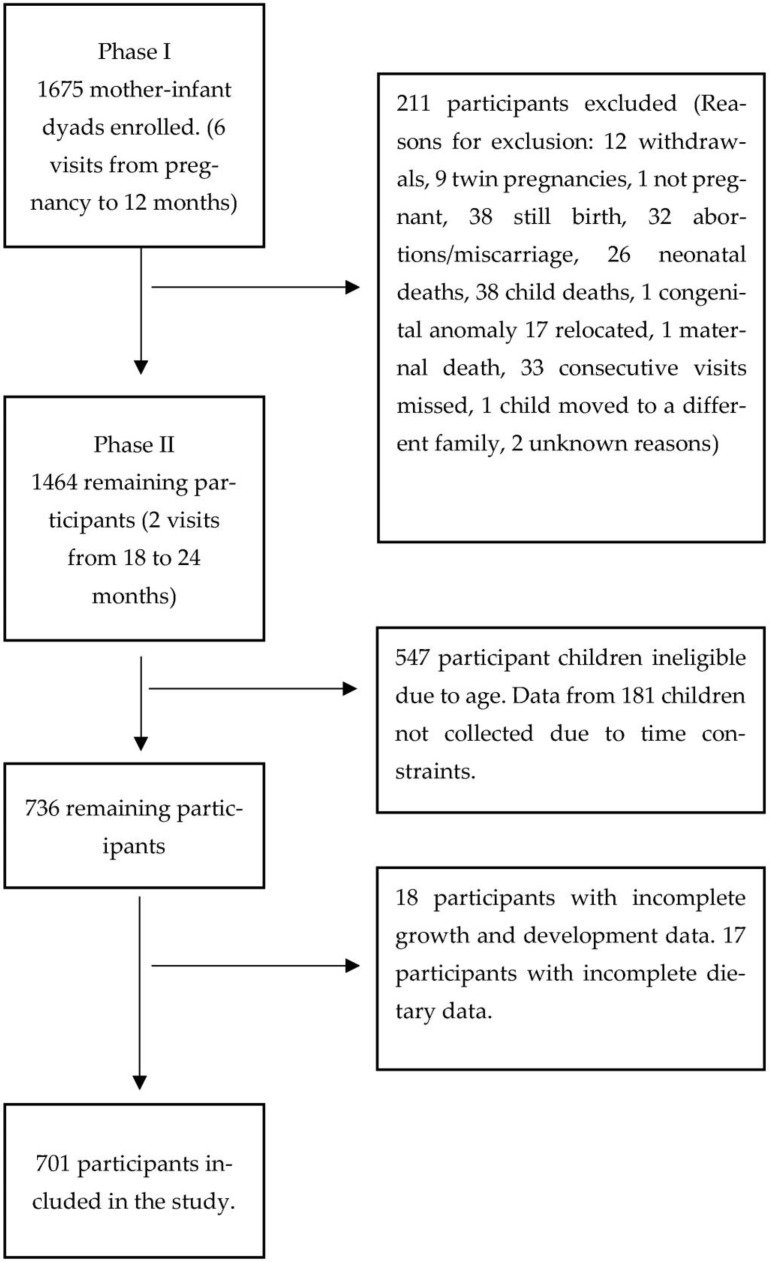
Study Participant flowchart.

We use data from the AflaCohort Study, an observational birth cohort study, conducted from 2015 to 2019 in Banke, Nepal. The latter ‘AflaCohort study’ examined relationships among mycotoxins exposure (Mycotoxins are natural, secondary metabolites produced by fungi species such as Aspergillus and Fusarium which are toxic to humans. For this study, exposure to Aflatoxin B1, Deoxynivalenol, and Fumonisin B1 was studied) and child growth and nutrition outcomes. In brief, 1675 pregnant women from a total of 17 village development committees (VDCs) (village development committee is a defunct district level administrative unit of Nepal. As per the Constitution of Nepal 2015, VDCs have been replaced by rural municipalities) were enrolled in the study. Eligible participants were healthy pregnant women 16 to 49 years of age, with a singleton pregnancy, <30 weeks of gestation at the time of enrollment, and intending to deliver and reside in the study area. Written informed consent was obtained from all participants. Ethical approval for the AflaCohort study was obtained from the Nepal Health Research Council (Registration no. 295/2014 and 199/2018) and from Tufts University’s Health Sciences Institutional Review Board (IRB#11535). The study was conducted in two phases (participant flowchart provided in Appendix A Figure A1). In the first phase, the mothers were assessed once during pregnancy, and later, the mother–child pairs were assessed at birth, 3, 6, 9, and 12 months after birth. In the second phase, two additional assessments of the mother and infant pairs were completed at infant age 18 to 22 months and 24 to 26 months (for simplicity, hereinafter referred to as 18 months and 24 months, respectively).

### 2.1. Data Collection

Data were collected by locally hired and trained enumerators using handheld Android tablets devices which included electronic, pre-coded data collection forms. Specific to this analysis, data extracted from the AflaCohort study database (all study data will be made publicly and freely available without restriction at https://data.usaid.gov/ (accessed on 11 November 2022) once all manuscripts related to the study’s original research questions have been published in peer-reviewed journals or upon request pending approval) included anthropometric data, developmental assessment results, and qualitative dietary recall reports.

### 2.2. Anthropometric Data

The anthropometric measurements of length, weight, and head circumference, collected at 24 months of age, were used in this analysis. Child length was measured to the nearest 0.1 cm using height–length ShorrBoard^®^ stadiometers. Weight was measured to the nearest 0.1 kg using Seca 874 digital scales. The child’s head circumference was measured to the nearest 0.1 cm using standard pediatric circumference bands. Three measurements were taken for each parameter and an average value was computed. The 2006 World Health Organization (WHO) growth standards were used to calculate anthropometric z-scores [34]. Children with length-for-age z-score (LAZ) between −2 and −3 were classified as moderately stunted and with LAZ below −3 were severely stunted. Similarly, children with weight-for-length z-score (WLZ) between −2 and −3 were classified as moderately wasted and below −3 as severely wasted. Similarly, children with head circumference-for-age z-score (HCZ) between −2 and −3 were classified as having moderate microcephaly and below −3 as having severe microcephaly. In accordance with WHO standards, one case with a LAZ outlier (i.e., LAZ < −6) was excluded in this analysis [34].

### 2.3. ECD Assessment

ECD was assessed at 24 months of age using the Ages-and-Stages Questionnaire (ASQ-3), a screening tool designed to assess developmental performance between the ages of 4 and 66 months [35,36]. The ASQ-3 has been used widely in various low-and middle-income country contexts [35,37,38,39]. In Nepal, the ASQ-3 has been used in both urban and rural community settings in Bhaktapur and Banke, respectively [24,25,40]. The ASQ is comprised of five domains with six questions per domain for a total of 30 questions. The five domains in the assessment are communication, gross motor, fine motor, problem-solving, and personal–social. The total range of possible scores for each domain is 0 to 60, giving the range of the total ASQ score to be 0 to 300. The questionnaire was translated and translated back to the locally spoken Nepali and Awadhi language. A minor adjustment to the questionnaire was made for cultural and contextual relevance and a question pertaining to the child using a fork to eat food was excluded and the domain score was adjusted according to the ASQ-3 guide [41]. For each question, there is a choice of three responses: ‘Yes’, ‘Sometimes’, or ‘Not Yet’ to represent the child’s ability to perform each task, with scores of 10, 5, and 0 assigned to each answer, respectively. Domain scores were calculated by adding the scores assigned to each answer. The total ASQ score was then calculated by adding the five domain scores.

Parent reporting is generally used for the ASQ assessments, but the ASQ-3 has been successfully used when administered by trained field workers [25,39]. For this study, four enumerators received didactic and practical training in developmental assessment methods by an experienced Nepali child development pediatrician at the Pediatric Department of the Institute of Medicine, Tribhuvan University, Nepal. A standard operating procedure was developed to guide the enumerators. Practical sessions were carried out by the pediatrician leading the training. Training assessment was carried out at the end of the training period by assessing the inter-rater reliability for five children where the pediatrician’s score served as the gold standard. We counted the missing responses by the enumerators as scores disagreeing. The inter-rater reliability percent agreement between the pediatrician and the four enumerators was 64%, 79%, 84%, and 90%. A trained research coordinator accompanied the field enumerators to the study area and remained with them as on-site supervisor for a total of 5 days and remained available for phone consultations during the data collection period. Age-appropriate modules of the ASQ assessments were used.

### 2.4. Child’s Diet and ASF Consumption

Information on the child’s diet was collected using a 24-h qualitative Food Frequency Questionnaire (FFQ) which was adapted from earlier studies in Nepal [42,43]. WHO minimum dietary diversity for children (MDD-C), a population level indicator to assess diet diversity among children aged 6 to 23 months, was calculated [44]. WHO guidelines were used to aggregate the individual food items to the following 4 food groups: (1) grains, roots, and tubers, (2) legumes and nuts, (3) vitamin-A rich fruits and vegetables, and (4) other fruits and vegetables [45]. Specific to this study’s objectives, 10 food items (namely, milk, yogurt, eggs, chicken, goat meat, buffalo meat, pork, large fish, small fish, and dried fish) were classified as the ASF group. We included multiple measures of any ASF consumption, as standards for assessing this measure have not yet been established. ASF consumption at 3 months was not used for computing ASF variables. The ASF variables computed include the following:

1. ASF consumption: defined as having consumed any ASF in the past 24 h (yes/no, binary variable).
2. Number of ASF consumed: defined as the total number of different ASF consumed in the past 24 h (possible range from 0 to 10). For instance, if a child consumed milk, meat, and eggs in the past 24 h, then the score equals 3.
3. ASF consumption frequency: defined as the total number of times each ASF was consumed by the child in the past 24 h. For instance, if a child consumed milk thrice and eggs once respectively in the past 24 h, then the score equals 4.
4. Cumulative number of ASF consumed: defined as the total number of ASF consumed in the past 24 h cumulated for all 4 time points (i.e., 6, 9, 12 and 18 months). For example, if for a child, the variable “number of ASF consumed” at each round of data collection was two, the cumulative number of ASF variable for that child is 8.
5. Cumulative ASF consumption frequency: defined as the total number of times each type of ASF consumed in the past 24 h cumulated for 4 time points. For example, if the variable “the ASF consumption frequency” at each time point was 4, the cumulative ASF consumption frequency variable is 16.

### 2.5. Other Characteristics and Variables

Several other variables were used or generated using the existing data, including household socio-economic characteristics, location, and women’s education and age (collected at enrollment). Household and socio-economic characteristics such as type of roof, floor, walls, toilet, cooking fuel, piped water, number of household members, and assets ownership were used to compute a household wealth index using Principal Component Analysis (PCA), a method that is used by Demographic Health Surveys (DHS) [46]. Households were classified into wealth index quintiles. Mother’s education was categorized into following groups: no schooling, primary schooling (1–5 years), secondary schooling (6–10 years), and more than secondary schooling (>10 years). Child’s sex, weight, and birth date were collected at birth. Child age in months at the 24-month visit was used in this analysis.

### 2.6. Statistical Analysis

This analysis is restricted to children for whom data on ASQ-3 assessment, HCZ and LAZ at 24 months, and ASF consumption from 6 to 18 months (all rounds) were available (*n* = 701). The main outcome variable of interest was total ASQ-3 score. HCZ, and LAZ at 24 months were other outcome variables that were investigated. The association with various past ASF consumption variables (as stated above) was tested in separate multivariable models.

Descriptive statistics were used to find the mean, standard deviation, and percentage of the outcome and control variables. Student’s t-tests or one-way ANOVA tests examined the differences in outcome variables by the categorical ASF variables. Similarly, Pearson correlation analysis examined the relationship between outcome variables and the continuous ASF variables. We used multivariable simple ordinary least squares (OLS) regression models to conduct a pooled analysis for each dependent variable controlling for the household wealth index, mother’s education, child’s sex, child’s age, and child’s food group consumption starting at 6 months of age, except for “grains, roots, and tuber”, which had very little variation in the later time points, as about 95%, 99%, and 100% of children consumed them at 9, 12, and 18 months, respectively. We examined the current breastfeeding status as a potential co-variate but did not include it in the models, as most children were breastfed across all time points with very little variation. All models were adjusted for clustering with the household location (i.e., VDC) used as a clustering variable. Analyses were performed using Stata SE 14.2. Significance levels for all analyses were set at a *p*-value lower than 0.05.

## 3. Results

### 3.1. Descriptive Characteristics

The general characteristics of the participant mothers and child is presented in Table 1, including child’s growth and ECD indicators at 24 months of child age.

### 3.2. Child Diet

At 3 months of age, about 73% of the children were reported to be exclusively breastfed. Continued breastfeeding was common, with 100%, 99%, 98%, and 91% of the children breastfed at 6 months, 9 months, 12 months, and 18 months, respectively. MDD-C increased with age as 32%, 45%, and 66% of children met the recommended 5 or more foods out of the 8 recommended food groups at 9 months, 12 months, and 18 months, respectively.

**Figure A2:**
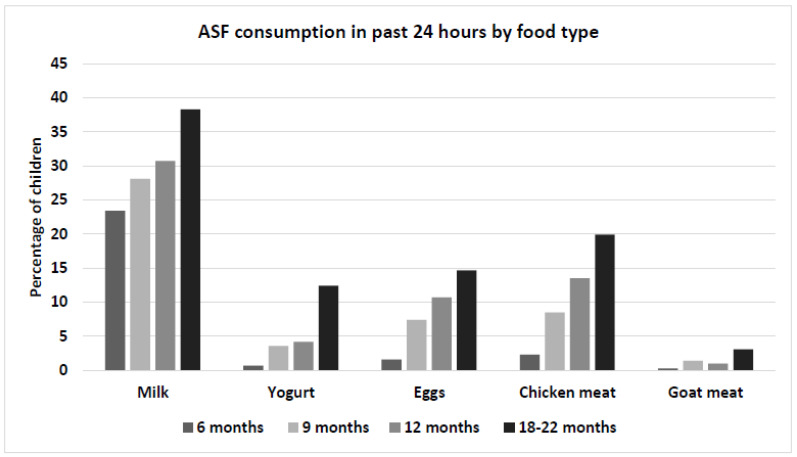
Type of ASF consumed by the child in the past 24 h.

Overall, ASF consumption was low in this sample. For the cumulative number of ASF consumed, the mean (SD) was 2.4 (2) ASF and the median was 2 ASF. For the cumulative ASF consumption frequency, the mean (SD) was 3.8 (3.7) times, and the median was 3 times. Child ASF consumption varied by age (Table 2); the percentage of children consuming ASF increased with child age. Likewise, the number of ASF consumed and the ASF consumption frequency increased with age. The different types of ASF consumed by the child by age is presented in Appendix A Figure A2. Milk was the most widely consumed ASF, with approximately 23%, 28%, 31%, and 38% of children consuming it in the past 24 h at 6 months, 9 months, 12 months, and 18 months, respectively. Chicken was the second most consumed ASF, followed by eggs. Very few children consumed any fish, yogurt, or other meats across all timepoints.

### 3.3. Bi-Variate Associations

The results of bi-variate tests between total ASQ score, HCZ, and LAZ and ASF consumption are shown in Appendix A Table A6. The ASQ score and LAZ were each positively associated with ‘any ASF’ consumption, the number of ASF, frequency of ASF consumed at all time points, and with cumulative ASF scores. HCZ had a significant positive association with any ASF consumption, number of ASF consumed, and frequency of ASF consumed at all time points except at 12 months. Likewise, HCZ had significant positive associations with the cumulative ASF scores. Bi-variate tests between the outcome variables show that the ASQ score was weakly correlated with HCZ (r = 0.23, *p* < 0.001) and moderately correlated with LAZ (r = 0.39, *p* < 0.001). HCZ and LAZ were both moderately correlated (r = 0.41, *p* < 0.001).

### 3.4. Relationship between ASQ Scores and ASF Consumption

The association between total ASQ scores and ASF consumption were examined in various multivariable models, controlling for LAZ and HCZ (Table 3). Total ASQ score was positively associated (β = 8.98, *p*-value < 0.01) with ASF consumption (yes/no) at 18 months. The relationship between ASQ scores and number of ASF consumed, ASF consumption frequency at different time points and cumulative ASF consumption were not statistically significant. LAZ had a strong, positive, and statistically significant association with ASQ scores across all models (Appendix A Table A1, Table A2, Table A3, Table A4 and Table A5).

### 3.5. Relationship between HCZ and ASF Consumption and LAZ and ASF Consumption

Next, we evaluated the relationship between HCZ and ASF consumption (Table 4). At 18 months, HCZ was positively associated (β = 0.24, *p*-value < 0.001) with ASF consumption (yes/no), with the number of ASF consumed (β = 0.13, *p*-value < 0.01) and with the ASF consumption frequency (β = 0.08, *p*-value < 0.01). The relationships between HCZ and the cumulative number of ASF consumed or the cumulative ASF consumption frequency were not statistically significant. Table 5 presents the association between LAZ and ASF consumption. Significant positive association (β = 0.11, *p*-value < 0.05) was observed between the LAZ and ASF consumption (yes/no) at 12 months. As reported in Zaharia et al. (2021), LAZ was found to be positively associated with both the cumulative number of ASF consumed (β = 0.06, *p*-value < 0.05) and the cumulative ASF consumption frequency (β = 0.03, *p*-value < 0.01).

## 4. Discussion

Our study finds significant positive association of total ASQ score with any ASF consumption at 18 months, controlling for LAZ and HCZ, suggesting the importance as well as the contribution of early life ASF consumption for better ECD outcomes, regardless of the nature of the relationship between ECD and physical growth. Even though we found significant bi-variate associations between ASQ scores and ASF variables such as number of ASFs, ASF consumption frequency at various time points of child age as well as cumulative consumption, these relationships seem to be attenuated by other factors in the long term. Nonetheless, the significant positive relationship between ASQ scores and any ASF consumption at 18 months, i.e., the time point closest to ASQ measurement, suggests that the presence of key nutrients such as protein, iron, zinc, vitamin B12, vitamin B2, vitamin B6, etc., in ASF, is crucial in brain function and cognitive development in early life [22,47]. The importance and contribution of ASF consumption in ECD is supported by many studies which have found similar associations [22,24,25,48]. Likewise, the significant strong positive relationship between ASQ scores and LAZ scores in all ASQ–ASF multivariable models show the interconnectedness between ECD outcomes and physical growth outcomes, and these findings are similar to that from past studies [10,13]. As discussed earlier, this association may be due to causal relationship between physical growth and ECD outcomes or a co-occurrence due to similar factors affecting them or a combination of both. In this study, we are unable to investigate the nature of the relationship between ECD outcomes and physical growth as we collected ECD outcomes only once at 24 months. We recommend future studies to explore this relationship further.

This study finds a significant positive association between LAZ score and cumulative ASF consumption (both number of ASF and ASF consumption frequency). This suggests that consistent ASF consumption in early life is important in linear growth of a child. In addition, there was a significant positive association between LAZ score and any ASF consumption at 12 months. Our findings support the findings from other studies which have found a significant positive association between LAZ and ASF consumption [30,32,33]. Several studies have also confirmed positive effect of ASF consumption on reduction of stunting [21,23,29].

Our study finds a positive association between HCZ with ASF consumption at 18 months including any ASF consumption, number of ASF consumed, and ASF consumption frequency. Although it is known that head circumference correlates well brain size, cognitive function, and learning. and that undernutrition is closely linked with microencephaly, few studies have studied the relationship of ASF consumption and head circumference [26,49]. The evidence on the relationship between ASF and HCZ remains mixed as a recent systematic review found that several randomized control trials did not find a significant relationship between head circumference outcomes and ASF consumption as complementary food/supplement [50]. However, several studies have found a significant relationship between ASF and head circumference. A Nepal study found that HCZ was related to the number of ASF consumed, with better HCZ in children who consumed two or more ASF compared to one or no ASF consumed [26]. In an observational longitudinal study in Nepal, Miller et al. (2020) found that milk consumption was also related to higher HCZ in children aged 24 to 60 months despite infrequent milk consumption. In a randomized control trial (RCT) on the first complementary food, infants aged 7 to 12 months consuming pureed beef had a higher rate of head circumference growth compared to infants consuming iron-fortified cereal [27]. Another RCT study on eggs supplementation found that children receiving eggs supplements had a higher HCZ compared to the control group [28]. More evidence is needed to fully understand the relationship between ASF consumption and head circumference.

This study finds poor child growth outcomes in the Banke district as there was a very high prevalence of stunting (>40%), high prevalence microcephaly (>30%), and high prevalence of wasting (>10%) among children aged 24 months. The level of stunting and wasting prevalence is higher than Nepal’s national prevalence of 36% and 10%, respectively [51]. The ECD outcomes, LAZ, and HCZ scores of this study differed from those found in other locations of Nepal [25,40,52]. This may be due to the differences in the socio-economic and community level characteristics of the population at these sites.

ASF consumption was found to be low at all time points, despite the percentage of children consuming ASF increasing gradually between 6 months to 18 months. There was a lack of variety in the types of ASF eaten, as indicated by the low mean and median scores of the number of ASF consumed. Milk was the most consumed ASF while the percentage of children consuming other ASF such as eggs, yogurt, meat, and poultry was below 20% at all time points. Likewise, the mean and median scores of the ASF consumption frequency were low at all time points suggesting that most children did not consume ASF often and consequently, the ASF quantity may have been insufficient to meet the nutrition requirements of the children. The mean and median scores of cumulative ASF consumption, calculated from all 4 time points of the study, were low. This is similar to the Miller et al. (2020) study which found the median cumulative score of all ASF groups, summed from 6 data collection points, to be low (i.e., 5 out of a possible score of 36). These findings suggest that there is a lack of variety in ASF consumption along with a sub-optimal quantity of ASF intake during the critical age of 6 to 18 months.

Overall, our findings suggest that ASF consumption is important in early life for good ECD outcomes. Even though cumulative ASF consumption and early life ASF consumption seem to be important, as suggested by the bi-variate results, their effect on ECD outcomes in the longer term seem to be attenuated when accounting for socio-economic and other related factors. This further supports the necessity of consistent ASF intake in children’s diet throughout early childhood, rather than just in certain periods in early life.

Our study has several strengths and limitations. In terms of strengths, this analysis was based on a large sample for which data on ASF consumption, ECD, and growth outcomes were all available at multiple time points. Thus, it was possible to investigate the associations between ASF consumption at several time points in early life and ECD outcomes at age 24 months, controlling for physical growth outcomes. Secondarily, we investigated the relationship between ASF consumption and physical growth. We could also examine the association between cumulative ASF consumption and ECD outcomes. As limitations, we could not collect ASQ data at multiple points. Hence, we could not examine the relationship between ASF consumption and ASQ scores longitudinally via panel data analysis and the relationships between ASF consumption in previous time points and ASQ scores were examined cross-sectionally. In regards to dietary data collection, there may also be a possibility of participant recall bias and social desirability bias when reporting the food consumed in the past 24 h. Similarly, we could not collect dietary data at 24 months due to budgetary and time constraints, and dietary data at 18 months were the closest estimate of the child’s diet during the time of ASQ data collection at 24 months.

## 5. Conclusions

Our findings highlight the importance of ASF in children’s diets for early childhood development and growth. Prior evidence also shows that ASF are rich in protein, energy-dense, and a good source of key micronutrients, all of which are essential for rapid development in early stages of life. Hence, our study recommends the need to support and promote ASF consumption at appropriate levels of intake among children through policy and programming actions on female education and behavior change; improvement in nutrition knowledge; improvement in livestock, poultry, and fish farming practices and production; improvement of market access and cold storage; and an overall increase in household income. Lack of refrigeration in households also seems to be a major constraint as ASF are perishable and most rural households do not have a quick access to the markets. Vegetarian food habits and religious beliefs also need to be accounted for when promoting ASF consumption among specific population groups. Addressing poor diet and ensuring sufficient ASF consumption are necessary steps in fulfilling the early childhood development and physical growth potential of young children in Nepal.

## Figures and Tables

**Table 1:** Child’s growth and development outcomes at 24 months and other characteristics.

	*n*	% or Mean (Standard Deviation)	Median (IQR)
Pre-term birth (<37 weeks of gestation)	65	9.3%	
Mean birth weight (kgs)	688	2.8 (0.45)	
Female child	365	52.1%	
Child ASQ total score	701	-	258 (218, 280)
Communication domain score		-	60 (50, 60)
Gross motor domain score		-	50 (30, 60)
Fine motor domain score		-	50 (40, 60)
Problem solving domain score		-	50 (40, 60)
Personal-social domain score		-	48 (48, 60)
Child LAZ score	700	−1.8 (1.1)	−1.8 (−2.6, −1.0)
Moderately stunted child (LAZ ≤ −2 and ≥−3)	211	30.1%	-
Severely stunted child (LAZ < −3)	93	13.3%	-
Child HCZ score	701	−1.7 (0.9)	−1.6 (−2.2, −1.0)
Moderate microcephaly (HCZ ≤ −2 and ≥−3)	193	27.5%	-
Severe microcephaly (HCZ < −3)	51	7.3%	-
Child WLZ score	696	−0.9 (1)	−0.9 (−1.5, −0.3)
Moderately wasted child (WLZ ≤ −2 and ≥−3)	76	11.0%	-
Severely wasted child (WLZ < −3)	13	1.8%	-
Maternal age (years)	701	26.3 (4.7)	
Mother’s schooling (%)			
No schooling	258	36.8	-
Primary (1–5 years)	149	21.3	-
Secondary (6–10 years)	235	33.5	-
More than Secondary (>10 years)	59	8.4	-
Household Wealth Quintile (%)			
Poorest	127	18.1	-
Poor	149	21.3	-
Middle	143	20.4	-
Rich	128	18.3	-
Richest	154	21.9	-

Notes: *n* denotes total frequency. LAZ refers to length-for-age z-score. WLZ refers to weight-for-length z-score. HCZ refers to head circumference-for-age z-score. ASQ refers to Ages and Stages questionnaire.

**Table 2:** Child’s ASF consumption by age.

	6 Months	9 Months	12 Months	18 Months
	Median (IQR) or %	Median (IQR) or %	Median (IQR) or %	Median (IQR) or %
Any ASF consumption	26.7%	40.1%	50.9%	63.9%
Number of ASF consumed	0 (0, 1)	0 (0, 1)	1 (0, 1)	1 (0, 1)
ASF consumption frequency	0 (0, 1)	0 (0, 1)	1 (0, 2)	1 (0, 2)
**Cumulative ASF consumption**				
Cumulative number of ASF consumed	-	-	-	2 (1, 4)
Cumulative ASF consumption frequency	-	-	-	3 (1, 5)

Notes: *n* = 701. IQR refers to interquartile range. Cumulative ASF consumption refer to cumulative ASF variables across all four time points.

**Table 3:** Association of total ASQ scores and ASF consumption.

Independent Variable	Multivariable Models				
	(1)	(2)	(3)	(4)	(5)
ASF consumption (Y/N)					
at 6 months	−0.45 (3.47)				
at 9 months	4.02 (2.59)				
at 12 months	−2.54 (3.99)				
at 18 months	8.98 ** (2.95)				
Number of ASF consumed					
at 6 months		−0.70 (3.16)			
at 9 months		1.69 (1.77)			
at 12 months		0.63 (2.22)			
at 18 months		2.89 (1.86)			
ASF consumption frequency					
at 6 months			−0.57 (1.33)		
at 9 months			1.60 (1.55)		
at 12 months			−1.50 (1.69)		
at 18 months			1.88 (1.18)		
**Cumulative consumption (6–18 months)**					
Number of ASF consumed				1.38 (0.68)	
Frequency of ASF consumption					0.30 (0.37)
***n***	700	700	700	700	700
***Adjusted R-square***	0.21	0.20	0.19	0.19	0.18

Notes: The table reports β coefficients and robust standard errors in parenthesis ** denotes *p*-value < 0.01. Models control for household wealth, mother’s education, child’s sex, child’s age, LAZ, HCZ, and child’s consumption of other food groups. Sensitivity analysis were carried out by including infant birthweight in all multivariable models and the results did not vary significantly. Cumulative consumption (6–18 months) refer to cumulative ASF variables across all four time points.

**Table 4:** Association of total HCZ scores and ASF consumption.

Independent Variable	Multivariable Models				
	(1)	(2)	(3)	(4)	(5)
ASF consumption (Y/N)					
at 6 months	0.02 (0.09)				
at 9 months	0.09 (0.08)				
at 12 months	−0.08 (0.06)				
at 18 months	0.24 *** (0.06)				
Number of ASF consumed					
at 6 months		0.06 (0.08)			
at 9 months		0.00 (0.06)			
at 12 months		−0.07 (0.05)			
at 18 months		0.13 ** (0.04)			
ASF consumption frequency					
at 6 months			−0.00 (0.03)		
at 9 months			0.03 (0.03)		
at 12 months			−0.06 (0.03)		
at 18 months			0.08 ** (0.03)		
**Cumulative consumption (6–18 months)**					
Number of ASF consumed				0.03 (0.02)	
Frequency of ASF consumption					0.01 (0.01)
***n***	701	701	701	701	701
***Adjusted R-square***	0.12	0.13	0.13	0.10	0.10

Notes: The table reports β coefficients and robust standard errors in parenthesis. *** denotes *p*-value < 0.001, ** denotes *p*-value < 0.01. Models control for household wealth, mother’s education, child’s sex, child’s age, and child’s consumption of other food groups. Cumulative consumption (6–18 months) refer to cumulative ASF variables across all four time points.

**Table 5:** Association of total LAZ scores and ASF consumption.

Independent Variable	Multivariable Models				
	(1)	(2)	(3)	(4)	(5)
ASF consumption (Y/N)					
at 6 months	0.06 (0.08)				
at 9 months	0.16 (0.09)				
at 12 months	0.11 * (0.05)				
at 18 months	0.13 (0.12)				
Number of ASF consumed					
at 6 months		0.03 (0.08)			
at 9 months		0.06 (0.05)			
at 12 months		0.06 (0.04)			
at 18 months		0.06 (0.06)			
ASF consumption frequency					
at 6 months			0.02 (0.04)		
at 9 months			0.05 (0.04)		
at 12 months			0.04 (0.02)		
at 18 months			0.03 (0.04)		
**Cumulative consumption (6–18 months)**					
Number of ASF consumed				0.06 * (0.02)	
Frequency of ASF consumption					0.03 ** (0.01)
***n***	700	700	700	700	700
***Adjusted R-square***	0.18	0.18	0.19	0.17	0.17

Notes: The table reports β coefficients and robust standard errors in parenthesis. ** denotes *p*-value < 0.01, * denotes *p*-value < 0.05. Models control for household wealth, mother’s education, child’s sex, child’s age, and child’s consumption of other food groups. Cumulative consumption (6–18 months) refer to cumulative ASF variables across all four time points.

## Data Availability

The data used in the present analysis can be obtained through request with the corresponding author.

## Appendix A

**Table A1:** Association of Total ASQ scores, HCZ, LAZ and daily consumption (Y/N) of all food groups.

	Total ASQ Score		HCZ Score		LAZ Score	
	β	Robust S.E.	β	Robust S.E.	β	Robust S.E.
Female child	0.71	(3.49)	−0.12	(0.06)	−0.03	(0.10)
Child age	−3.51	(2.59)	0.00	(0.04)	0.03	(0.04)
Mother’s schooling (REF: None)						
Primary	6.84	(6.83)	−0.05	(0.08)	0.23 *	(0.09)
Secondary	9.93	(4.98)	0.08	(0.09)	0.60 ***	(0.12)
More than Secondary	11.65	(6.97)	0.37 *	(0.13)	0.78 ***	(0.13)
Household Wealth Quintile (REF: Poorest)						
Poor	−2.59	(6.98)	−0.01	(0.07)	0.05	(0.14)
Middle	0.67	(5.50)	0.00	(0.07)	0.03	(0.09)
Rich	3.07	(5.26)	0.27 *	(0.12)	0.07	(0.12)
Richest	4.46	(6.53)	0.16	(0.09)	−0.16	(0.10)
LAZ at 24 months	14.11 ***	(1.98)	-	-	-	-
HCZ at 24 months	1.40	(1.89)	-	-	-	-
Daily legumes and nuts consumption (Y/N)						
at 6 months	−0.91	(4.02)	0.19	(0.10)	0.12	(0.10)
at 9 months	0.29	(4.65)	−0.01	(0.09)	−0.03	(0.07)
at 12 months	9.51	(4.79)	0.03	(0.11)	−0.18	(0.12)
at 18 months	4.85	(5.68)	−0.05	(0.08)	0.05	(0.10)
Daily vitamin A rich food consumption (Y/N)						
at 9 months	−0.97	(2.54)	0.21 *	(0.08)	0.17 *	(0.06)
at 12 months	−0.45	(4.92)	−0.06	(0.06)	0.15	(0.11)
at 18 months	−1.91	(4.21)	0.03	(0.05)	0.15 *	(0.06)
Daily other fruits and vegetables consumption (Y/N)						
at 6 months	−0.16	(4.84)	0.08	(0.05)	0.10	(0.08)
at 9 months	2.25	(2.86)	0.08	(0.05)	0.07	(0.07)
at 12 months	0.94	(3.29)	0.02	(0.09)	0.21	(0.12)
at 18 months	13.50 *	(5.49)	0.10	(0.08)	0.00	(0.06)
Daily ASF consumption (Y/N)						
at 6 months	−0.45	(3.47)	0.02	(0.09)	0.06	(0.08)
at 9 months	4.02	(2.59)	0.09	(0.08)	0.16	(0.09)
at 12 months	−2.54	(3.99)	−0.08	(0.06)	0.11 *	(0.05)
at 18 months	8.98 **	(2.95)	0.24 ***	(0.06)	0.13	(0.12)
*n*	700		701		700	
*Adjusted R-square*	0.21		0.12		0.18	

Note: β denotes the coefficient. Robust S.E. denotes Robust standard errors. *n* denotes the total frequency. REF denotes the reference group of comparison. ASQ refers to Ages and Stages questionnaire. HCZ refers to Head circumference for age Z-scores. LAZ refers to Length for age Z-scores. *** denotes *p*-value < 0.001, ** denotes *p*-value < 0.01, * denotes *p*-value < 0.05. Daily grains and tubers consumption variables are not included in the models as most children have consumed this food group at 9 months, 12 months, and 18 months of age. Daily vitamin A rich foods consumption at 6 months is not included in the model as only 3% of children had consumed it. Vitamin A rich food also includes consumption of dark green leafy vegetables.

**Table A2:** Association between ASQ score, HCZ and LAZ, and the daily number of food groups consumed.

	Total ASQ Score		HCZ Score		LAZ Score	
	β	Robust S.E.	β	Robust S.E.	β	Robust S.E.
Female child	0.30	(3.42)	−0.12	(0.06)	−0.03	(0.10)
Child age	−3.20	(2.78)	0.01	(0.04)	0.03	(0.04)
Mother’s schooling (REF: None)						
Primary	6.70	(7.23)	−0.08	(0.07)	0.18	(0.09)
Secondary	10.12	(5.03)	0.06	(0.09)	0.56 ***	(0.12)
More than Secondary	11.35	(7.71)	0.35 *	(0.13)	0.76 ***	(0.13)
Household Wealth Quintile (REF: Poorest)						
Poor	−2.38	(7.01)	−0.02	(0.08)	0.05	(0.14)
Middle	0.92	(5.23)	−0.02	(0.06)	0.04	(0.10)
Rich	2.40	(4.56)	0.23 *	(0.11)	0.04	(0.13)
Richest	5.19	(6.26)	0.17	(0.08)	−0.15	(0.10)
LAZ at 24 months	14.00 ***	(2.10)	-	-	-	-
HCZ at 24 months	1.80	(1.92)	-	-	-	-
Daily number of grains and tubers consumed						
at 6 months	3.60	(3.07)	−0.06	(0.06)	−0.03	(0.06)
at 9 months	−0.21	(2.49)	−0.03	(0.04)	−0.07	(0.05)
at 12 months	0.47	(2.01)	−0.02	(0.03)	0.03	(0.04)
at 18 months	−0.15	(0.20)	0.01	(0.01)	0.01 ***	(0.00)
Daily number of legumes and nuts consumed						
at 6 months	−4.96	(3.38)	0.14	(0.09)	0.09	(0.09)
at 9 months	0.87	(2.90)	−0.02	(0.05)	0.06	(0.03)
at 12 months	5.33	(2.80)	0.07	(0.07)	−0.06	(0.06)
at 18 months	0.43	(1.85)	−0.00	(0.03)	0.03	(0.05)
Daily number of vitamin A rich food consumed						
at 9 months	−1.56	(2.20)	0.21 *	(0.07)	0.12	(0.06)
at 12 months	−0.48	(4.36)	−0.07	(0.07)	0.12	(0.10)
at 18 months	−2.07	(3.52)	−0.00	(0.04)	0.11	(0.05)
Daily number of other fruits and vegetables consumed						
at 6 months	−0.74	(3.35)	0.06	(0.04)	0.03	(0.06)
at 9 months	0.01	(1.73)	0.05	(0.03)	0.10	(0.05)
at 12 months	−1.21	(1.94)	0.04	(0.04)	0.06	(0.04)
at 18 months	2.84	(1.50)	0.04	(0.02)	0.04	(0.02)
Daily number of ASF consumed						
at 6 months	−0.70	(3.16)	0.05	(0.08)	0.03	(0.08)
at 9 months	1.69	(1.77)	0.01	(0.06)	0.06	(0.05)
at 12 months	0.63	(2.22)	−0.07	(0.05)	0.06	(0.04)
at 18 months	2.89	(1.86)	0.13 **	(0.04)	0.06	(0.06)
*n*	700		701		700	
*Adjusted R-square*	0.20		0.13		0.18	

Note: β denotes the coefficient. Robust S.E. denotes Robust standard errors. *n* denotes the total frequency. REF denotes the reference group of comparison. ASQ refers to Ages and Stages questionnaire. HCZ refers to Head circumference for age Z-scores. LAZ refers to Length for age Z-scores. *** denotes *p*-value < 0.001, ** denotes *p*-value < 0.01, * denotes *p*-value < 0.05. Daily vitamin A rich foods consumption at 6 months is not included in the model as only 3% of children had consumed it. Vitamin A rich food also includes consumption of dark green leafy vegetables.

**Table A3:** Association between ASQ score, HCZ and LAZ, and the daily food group consumption frequency.

	Total ASQ Score		HCZ Score		LAZ Score	
	β	Robust S.E.	β	Robust S.E.	β	Robust S.E.
Female child	0.43	(3.32)	−0.10	(0.06)	−0.02	(0.10)
Child age	−3.04	(2.85)	0.01	(0.04)	0.02	(0.04)
Mother’s schooling (REF: None)						
Primary	7.05	(7.15)	−0.09	(0.08)	0.19 *	(0.09)
Secondary	11.71 *	(4.87)	0.04	(0.09)	0.55 ***	(0.13)
More than Secondary	15.07	(7.73)	0.38 **	(0.13)	0.75 ***	(0.11)
Household Wealth Quintile (REF: Poorest)						
Poor	−1.97	(7.42)	−0.00	(0.08)	0.04	(0.13)
Middle	1.33	(5.22)	−0.02	(0.06)	0.05	(0.09)
Rich	3.88	(4.75)	0.25 *	(0.11)	0.03	(0.12)
Richest	7.27	(6.48)	0.18 *	(0.07)	−0.14	(0.09)
LAZ at 24 months	14.34 ***	(2.20)	-	-	-	-
HCZ at 24 months	1.81	(2.06)	-	-	-	-
Daily grains and tubers consumption frequency						
at 6 months	0.12	(1.09)	−0.01	(0.03)	0.01	(0.03)
at 9 months	0.05	(1.06)	−0.00	(0.02)	−0.00	(0.03)
at 12 months	−0.38	(0.87)	−0.03	(0.02)	0.01	(0.03)
at 18 months	0.31	(1.06)	−0.01	(0.01)	−0.05 **	(0.02)
Daily legumes and nuts consumption frequency						
at 6 months	−1.49	(1.80)	0.07	(0.05)	0.06	(0.05)
at 9 months	0.45	(1.92)	0.01	(0.03)	0.03	(0.03)
at 12 months	0.87	(1.40)	0.02	(0.04)	−0.04	(0.03)
at 18 months	0.19	(0.93)	−0.02	(0.02)	−0.00	(0.03)
Daily vitamin A rich food consumption frequency						
at 6 months	−2.54	(6.41)	0.30 **	(0.10)	0.22	(0.18)
at 9 months	0.32	(1.82)	0.13	(0.06)	0.10	(0.05)
at 12 months	1.94	(2.64)	−0.06	(0.04)	0.06	(0.06)
at 18 months	−1.20	(1.46)	−0.00	(0.02)	0.08 *	(0.03)
Daily other fruits and vegetables consumption frequency						
at 6 months	1.42	(1.28)	0.03 *	(0.01)	−0.01	(0.03)
at 9 months	0.93	(1.36)	0.04	(0.02)	0.07	(0.04)
at 12 months	−1.28	(1.32)	0.03	(0.03)	0.03	(0.03)
at 18 months	1.24	(1.08)	0.04 *	(0.01)	0.03	(0.01)
Daily ASF consumption frequency						
at 6 months	−0.57	(1.33)	−0.00	(0.03)	0.02	(0.04)
at 9 months	1.60	(1.55)	0.03	(0.03)	0.05	(0.04)
at 12 months	−1.50	(1.69)	−0.06	(0.03)	0.04	(0.02)
at 18 months	1.88	(1.18)	0.08 **	(0.03)	0.03	(0.04)
*n*	700		701		700	
*Adjusted R-square*	0.19		0.13		0.19	

Note: β denotes the coefficient. Robust S.E. denotes Robust standard errors. *n* denotes the total frequency. REF denotes the reference group of comparison. ASQ refers to Ages and Stages questionnaire. HCZ refers to Head circumference for age Z-scores. LAZ refers to Length for age Z-scores. *** denotes *p*-value < 0.001, ** denotes *p*-value < 0.01, * denotes *p*-value < 0.05. Vitamin A rich food also includes consumption of dark green leafy vegetables.

**Table A4:** Association between ASQ score, HCZ and LAZ, and the cumulative total of the number of food groups consumed.

	Total ASQ Score		HCZ Score		LAZ Score	
	β	Robust S.E.	β	Robust S.E.	β	Robust S.E.
Female child	0.74	(3.20)	−0.10	(0.06)	−0.02	(0.09)
Child age	−2.97	(2.72)	0.01	(0.04)	0.02	(0.03)
Mother’s schooling (REF: None)						
Primary	7.73	(6.79)	−0.08	(0.07)	0.18	(0.09)
Secondary	10.07	(5.13)	0.06	(0.09)	0.56 ***	(0.12)
More than Secondary	11.14	(7.38)	0.37 **	(0.12)	0.76 ***	(0.13)
Household Wealth Quintile (REF: Poorest)						
Poor	−2.67	(7.10)	0.02	(0.09)	0.05	(0.13)
Middle	0.47	(5.35)	0.01	(0.06)	0.05	(0.09)
Rich	2.91	(4.62)	0.27 *	(0.12)	0.04	(0.12)
Richest	5.62	(6.01)	0.19 *	(0.07)	−0.15	(0.10)
LAZ at 24 months	13.73 ***	(1.99)	-	-	-	-
HCZ at 24 months	1.96	(1.79)	-	-	-	-
Cumulative total of the number of grains and tubers consumed	−0.08	(0.16)	0.00	(0.01)	0.01 *	(0.00)
Cumulative total of the number of legumes and nuts consumed	1.26	(1.13)	0.03	(0.02)	0.01	(0.02)
Cumulative total of the number of vitamin A rich foods consumed	−1.44	(1.79)	0.06 *	(0.02)	0.12 ***	(0.03)
Cumulative total of the number of other fruits and vegetables consumed	0.94	(0.78)	0.04 *	(0.01)	0.05 **	(0.01)
Cumulative total of the number of ASFs consumed	1.38	(0.68)	0.03	(0.02)	0.06 *	(0.02)
*n*	700		701		700	
*Adjusted R-square*	0.19		0.10		0.17	

Note: β denotes the coefficient. Robust S.E. denotes Robust standard errors. *n* denotes the total frequency. REF denotes the reference group of comparison. ASQ refers to Ages and Stages questionnaire. HCZ refers to Head circumference for age Z-scores. LAZ refers to Length for age Z-scores. *** denotes *p*-value < 0.001, ** denotes *p*-value < 0.01, * denotes *p*-value < 0.05. Vitamin A rich food also includes consumption of dark green leafy vegetables.

**Table A5:** Association between ASQ score, HCZ and LAZ, and the cumulative total of the food group consumption frequency.

	Total ASQ Score		HCZ Score		LAZ Score	
	β	Robust S.E.	β	Robust S.E.	β	Robust S.E.
Female child	0.43	(3.23)	−0.11	(0.06)	−0.03	(0.10)
Child age	−3.00	(2.72)	0.01	(0.04)	0.02	(0.03)
Mother’s schooling (REF: None)						
Primary	7.86	(6.82)	−0.08	(0.07)	0.19 *	(0.09)
Secondary	11.47 *	(4.97)	0.09	(0.09)	0.59 ***	(0.12)
More than Secondary	14.02	(7.36)	0.42 **	(0.12)	0.78 ***	(0.10)
Household Wealth Quintile (REF: Poorest)						
Poor	−2.50	(7.14)	0.02	(0.09)	0.04	(0.13)
Middle	1.13	(5.25)	0.04	(0.07)	0.07	(0.09)
Rich	2.94	(4.75)	0.27 *	(0.12)	0.02	(0.12)
Richest	6.56	(6.27)	0.20 **	(0.06)	−0.14	(0.09)
LAZ at 24 months	13.95 ***	(2.08)				
HCZ at 24 months	2.19	(1.83)				
Cumulative total of the grains and tubers consumption frequency	−0.02	(0.42)	−0.01	(0.01)	−0.01	(0.01)
Cumulative total of the legumes and nuts consumption frequency	0.34	(0.66)	0.02	(0.01)	0.01	(0.02)
Cumulative total of the vitamin A rich foods consumption frequency	−0.18	(0.88)	0.03	(0.02)	0.08 **	(0.02)
Cumulative total of other fruits and vegetables consumption frequency	0.51	(0.60)	0.04 ***	(0.01)	0.04 **	(0.01)
Cumulative total of ASFs consumption frequency	0.30	(0.37)	0.01	(0.01)	0.03 **	(0.01)
*n*	700		701		700	
*Adjusted R-square*	0.18		0.09		0.17	

Note: β denotes the coefficient. Robust S.E. denotes Robust standard errors. *n* denotes the total frequency. REF denotes the reference group of comparison. ASQ refers to Ages and Stages questionnaire. HCZ refers to Head circumference for age Z-scores. LAZ refers to Length for age Z-scores. *** denotes *p*-value < 0.001, ** denotes *p*-value < 0.01, * denotes *p*-value < 0.05. Vitamin A rich food also includes consumption of dark green leafy vegetables.

**Table A6:** Bivariate associations of ASQ, HCZ, and LAZ with ASF consumption.

	Total ASQ Score		HCZ Score		LAZ Score	
	Mean Score or Correlation Coefficient	*p*-Value	Mean Score or Correlation Coefficient	*p*-Value	Mean Score or Correlation Coefficient	*p*-Value
Any ASF consumption (Y/N) ^a^						
at 6 months	Y: 254 N: 241	0.0024	Y: −1.44 N: −1.73	0.0003	Y: −1.49 N: −1.91	0.0000
at 9 months	Y: 253 N: 239	0.0002	Y: −1.52 N: −1.73	0.0029	Y: −1.54 N: −1.97	0.0000
at 12 months	Y: 249 N: 240	0.0229	Y: −1.59 N: −1.71	0.1073	Y: −1.63 N: −1.97	0.0000
at 18 months	Y: 251 N: 234	0.0000	Y: −1.53 N: −1.86	0.0000	Y: −1.69 N: −1.99	0.0006
Number of ASFs consumed ^b^						
at 6 months	0.10	0.0067	0.14	0.0002	0.16	0.0000
at 9 months	0.14	0.0001	0.11	0.0026	0.19	0.0000
at 12 months	0.12	0.0010	0.06	0.0759	0.17	0.0000
at 18 months	0.17	0.0000	0.19	0.0000	0.17	0.0000
ASF consumption frequency ^b^						
at 6 months	0.09	0.0201	0.09	0.0156	0.16	0.0000
at 9 months	0.13	0.0004	0.10	0.0055	0.19	0.0000
at 12 months	0.08	0.0354	0.04	0.2492	0.17	0.0000
at 18 months	0.13	0.0004	0.17	0.0000	0.14	0.0002
Cumulative Consumption (6–18 months) ^b^						
Number of ASF consumed	0.21	0.0000	0.19	0.0000	0.26	0.0000
Frequency of ASF consumption	0.16	0.0000	0.14	0.0001	0.24	0.0000
*n*	701		701		700	

Note: *n* denotes the total frequency. ^a^ t tests were carried out to check the equality of means. ^b^ pairwise correlation was carried out to check for correlation.
